# The Impact of U.S. Housing Type and Residential Living Situations on Mental Health during COVID-19

**DOI:** 10.3390/ijerph18168281

**Published:** 2021-08-05

**Authors:** Jyotsna Ghimire, Andrew T. Carswell, Ramesh Ghimire, Pamela R. Turner

**Affiliations:** 1 Department of Financial Planning, Housing, and Consumer Economics, University of Georgia, Athens, GA 30602, USA; jyotsna.ghimire@uga.edu (J.G.); prturner@uga.edu (P.R.T.); 2 Warnell School of Forestry and Natural Resources, University of Georgia, Athens, GA 30602, USA; ghimire@uga.edu

**Keywords:** mental health, housing, apartments, property management, COVID-19

## Abstract

Residential environments could be associated with the mental health of residents, in general, and during the COVID-19 pandemic. However, limited studies have investigated the relationship between these two. This study used data from the Household Pulse Survey, collected between 23 April 2020 and 23 November 2020 to explore the relationship between mental health status as perceived by the residents and housing tenure (own or rent), building type, and the number of household members, while accounting for sociodemographic characteristics, general health-related variables, and week-specific unobserved heterogeneities. The findings suggest that renters had higher odds of experiencing mental health issues than homeowners. Residents in multifamily housing units had higher odds of experiencing mental health problems than single-family units. Further, more people in the household were associated with lower odds of experiencing mental health episodes during the COVID-19 pandemic.

## 1. Introduction

The COVID-19 pandemic has impacted several aspects of life, with one being the home environment. Apart from disaster scenarios which require evacuations, the pandemic has required most Americans to shelter in place rather than retreat from an external threat. [1] stated that “[t]he COVID-19 pandemic incorporates a suite of health, economic and political challenges; housing is emerging as one of them” (p. 177). Due to shelter-in-place orders enforced by various government agencies, numerous people were confined to their homes for many weeks. Due to differences in residential environments, these orders could have exacerbated residents’ mental health problems.

While rental communities are perceived to be more adversely affected by the pandemic than homeowners [2], limited studies have investigated the relationship between residential environments and residents’ mental health during the pandemic. Using data from the Household Pulse Survey (HPS), conducted between 23 April and 23 November 2020, this study explored the relationship between residential environments and mental health status as perceived by residents during the COVID-19 pandemic. The relationship between the mental health status of residents and their housing tenure (own or rent) and household size while controlling for their sociodemographic characteristics, general health-related variables, and week-specific unobserved heterogeneities were examined. Then, the housing tenure variable was replaced by building type, and the relationship between these two was examined.

### Residential Environments and Mental Health during Pandemic

Residential environments can be an important factor in predicting mental health status in general and during the COVID-19 pandemic. Multifamily homes provide unique situations that create complexity for residents. Normally thought of as a respite from crises, apartment homes can foster additional anxiety among residents. Research has shown adverse psychological consequences in high-density housing situations independent of pandemics [3,4]. These high-density environments make social distancing difficult to maintain. In several U.S. metropolitan areas, the share of households residing in apartment buildings either meets or exceeds 20 percent [5]. Many U.S. renters have overextended their budgets beyond their ability to pay for rental units, which is commonly defined as 30 percent or less of gross income [6]. For those who are already low-income, rental affordability has become a larger problem during the pandemic. Because renters have demonstrably fewer savings than their home-owning counterparts [7], the pandemic has potentially dire economic consequences, especially for low-income households [8]. Homeowners may have more resources (e.g., home equity) to cope with financial hardship than renters [6].

These affordability problems have also been shown to have negative effects on households’ mental health [9]. Due to the potential fragility of many renters’ situations, public health experts identified those with “precarious” housing situations as highly vulnerable populations during the pandemic, which necessitates the need for multidisciplinary research efforts [10].

Including the public health and economic concerns that the COVID-19 pandemic has engendered, mental health aspects have recently emerged [11]. The COVID-19 effect on certain housing situations could exacerbate already existing mental health conditions. There has been a pronounced lack of research on the intersection of housing situations and household mental health, although there is evidence that higher-density apartment living does create strains on psychological well-being even during pre-pandemic periods [12]. Furthermore, recent research shows the effect that subsidized housing has on adolescent mental health, relative to its private sector counterparts [13]. Property management personnel are not normally well-versed in recognizing or responding to tenants’ mental health issues, but the topic is currently being discussed within the profession as the pandemic has lingered over many months [14]. In mid-2020, the [15] released its best practices for addressing residents with mental health concerns. These mental health matters can be exacerbated by the identification of a positive virus case within the building, which can increase anxiety over who will be next to catch the virus [14]. Domestic violence also becomes a concern for many as they become subject to lockdown procedures and loss of income [16,17,18]. The threat of eviction due to economic circumstances can also have effects on mental health. [19] found evidence of mental health situations during normal eviction situations before COVID-19. These mental health issues can be more pronounced for children, even several years after the eviction event [20]. Household financial crises which are not tied to evictions have also been proven to cause psychological distress [21,22]. Because the economic impact of COVID-19 has most disproportionately affected lower-income Americans [23] and because most apartment dwellers are classified as low- to moderate-income, it is possible that the multifamily housing industry is primed for a mental health crisis due to the fallout from COVID-19.

## 2. Data and Methods

### 2.1. Data Source

This analysis was conducted using the U.S. Census Bureau’s Household Pulse Survey, a national-level household survey, collected between 23 April and 23 November 2020. The Household Pulse Survey was a weekly 20-minute online survey, conducted by U.S. Census Bureau in collaboration with other federal agencies. The Household Pulse Survey was designed to complement the ability of the federal statistical system to rapidly respond and provide relevant information about the scope of the impact of COVID-19 in the US [24]. The survey covers questions about childcare, education, employment, food security, health, housing, social security benefits, household spending, consumer spending associated with stimulus payments, intention to receive a COVID-19 vaccination, and transportation affected by the ongoing crisis [25]. Hence, the survey collects information on how the coronavirus pandemic has been socially and economically impacting households across the country. The Census Bureau randomly chooses the address of the household across the country to ensure the representation of the entire population. However, households’ participation in the survey is completely voluntary [25].

The survey’s noteworthy variables that have been used within the various analyses are shown in Table 1. Many of these variables included control variables that related to the various demographic and socioeconomic characteristics of the households (age, race, income, gender, education level, and marital status). Four of the binary variables within the survey (Down, Anxious, Worry, and Interest) indicated the degree to which the household was suffering from mental health concerns, particularly as they related to feeling down, experiencing anxiety, being worried, and losing interest. Based on these four variables, a binary indicator variable for mental health was constructed. The variable mental health equals one if the survey respondent indicated that they felt down (several days, more than half the days, nearly every day) or experienced anxiety (several days, more than half the days, nearly every day) or was worried (several days, more than half the days, nearly every day), or lost interest (several days, more than half the days, nearly every day), and zero otherwise.

Housing environments can play an important role in coping with this pandemic. The three home-specific variables that were tracked for effects on participants’ mental health were the number of people residing within the home, homeowner versus renter status, and building type. The expectations were that the number of people within the home would have a positive relationship with mental health concerns, as the greater number of people provided potentially more exposure to infection for someone who was a carrier. Added to that, more people within a finite amount of space created less privacy and undoubtedly more stress. Homeowners were expected to have less stress for many of these same privacy concerns. Whereas homeowners were more likely to have more square footage within their dwelling unit, there was also a sense of control within homeownership situations that was more empowering and less stressful than a rental situation. The anxieties within apartment scenarios were illustrative of that reality. Even though property management is a growing profession with certain sets of industry care and standards, it is not clear whether all property managers are consistent in providing quality across the board. Simultaneously, these managers have a large degree of control over the various policies of that building, not the least of which includes who can enter (including those unknowingly carrying the virus). Such lack of control could detrimentally impact one’s mental health. Thus, the building type was expected to have a positive relationship with mental health concerns as building density increased, for many of the same reasons stated for renters in general but also including social distancing concerns.

Still, another set of variables captured mitigating circumstances that could impact and exacerbate the mental health concerns of household members, many of which were specific to the pandemic. These included such things as loss of job (both expected and actual), health insurance availability, and health status. Food availability was also captured and included within the model, both from an actual standpoint and relative to what the food supply was before the pandemic. As people were both reluctant to travel to grocery stores and store inventories for various food items began to dwindle during the spring and early summer, this change in buyer behavior was likely to cause anxiety among households. Many, if not all, of these variables could logically be considered an offshoot of the pandemic itself. Also, a separate set of variables was isolated that identified the housing-specific characteristics of the household as it related to housing tenure, type of housing unit, and number of people living within the unit. There was a temporal variable included within the model, as time spent dealing with the coronavirus may indeed affect people. The COVID-19 pandemic provided a unique situation in which households were confined to their living quarters, sometimes for extended periods of time. The Household Pulse Survey was cross-sectional and covered many households per week. A snapshot of the distribution of responses for each of the initial 19 weeks covered by the survey has been provided in Table 2. On average, the survey covered over 80,481 U.S. households per week, with a total of over 1.5 million households surveyed over the 19-week period.

The descriptive statistics on Table 3 showed that the Household Pulse Survey respondents represented a wide swath of the U.S. population. Most of the survey respondents had household heads aged 31–60, with nearly a third being over 60, which is largely considered the most vulnerable age group for the virus. The rest of the demographic profile showed that most respondents were white, married, females who have at least a college degree. While the most popular answer for household size was two people, over 13% of the respondents had households of five or more people. Nearly 17% of the Pulse respondents lived alone, a section of the population that could face real challenges in the increasing isolation brought on by the pandemic.

Mental health statistics show the damaging toll that the pandemic has on the general population and highlights a hidden cost of the pandemic. The responses to each of the four mental health variables of interest suggested a public health concern beyond that of contracting COVID-19. Nearly two-thirds of the country experienced a mental health concern of some nature at the onset of the pandemic [25]. While the percentages seemed to subside steadily over time, there was a spike in the percentage of mental health concerns in Week 13 of the Household Pulse Survey, between the dates of 19 August and 31. Generally speaking, however, the passage of time alone (aside from the spike in Week 13) did not seem to cause a spike in overall mental health concerns. The slight downturn from week to week suggested that families may have figured out ways to cope with the pandemic. Still, the percentages remained high by the last reporting period (Week 19). Likewise, the mental health concerns were higher for renters than homeowners by almost ten percentage points throughout the study period. These percentages were reported in Table 4A.

Table 4B reports the mental health concerns during the pandemic (since week 13) by living arrangement. Although the mental health concerns grew over weeks, the mental health concerns were higher for those who live in medium density (2–9 units) or higher density units (10 or more), as compared to those who live in low-density units (single-family units).

### 2.2. Econometric Model

This study hypothesized that the mental health status of an individual ‘i’ (M_i_) depends on his/her residential environments (**R_i_**), while controlling for his/her sociodemographic characteristics (**S**_i_) and week-specific unobserved heterogeneities (**W**_k_). This relationship was expressed in Equation (1).

M_i_ = f(**R**_i_; **S**_i_; **W**_k_) (1)

The residential environments (**R**), the variable of interest, include housing tenure (own or rent) or building type, and number of members in the household. The sociodemographic characteristics (**S**) which were used in this analysis included household income, race/ethnicity, education, marital status, number of children in the household, gender, work loss, any work, food security status, health status, and health insurance status (Table 1).

The week-fixed effects (**W**) were used to account for week-specific unobserved heterogeneities that affected the dependent or independent variables in the model. For instance, people may have experienced stress on mental health differently based on weeks during the state-wide lockdown. People may have alternated between employment and unemployment from week to week. The use of week-fixed effects (**W**) mitigated the influence of such events in the regression analysis.

Since the mental health status of an individual was modeled as a binary response, logistic regression was used to estimate the model (Equation (1)). Equation (2) summarized the logistic regression model.

(2) $$ln\frac{P\left(Y_{i}=1\right)}{1-P\left(Y_{i}=1\right)}=\ln\left(Odds_{i}\right)=\alpha +\sum_{k=1}^{K}\beta_{k}X_{ik}=\alpha +\beta \;X$$

In the equation above, X was the vector of explanatory variables, including the variables of interests, week-fixed effects, and controls (**S**, R, and W) and β was the vector of parameters to be estimated [26]. R programming language was used to estimate the model. Since logistic regression coefficients do not correspond to marginal effects as in the linear regression model, odds ratios were computed by exponentiating the logistic regression coefficients to ease the interpretation of the regression results.

## 3. Results

### 3.1. Mental Health and Residential Environment: Housing Tenure and Number of Household Member

Table 5 reported logistic regression coefficients and odds ratios for the full sample (Weeks 1–19), with housing tenure status and the number of household members as key explanatory variables. Rental residents experienced more mental health problems than their owning counterparts, after controlling for all other variables. As shown in Table 5, the odds of having mental health problems for people who live in rental properties were 1.172 times those who own their homes. While many homeowners still must pay monthly obligations, others are either far along in their loan obligations or own their homes free and clear, thus reducing if not nullifying the fear of being kicked out of their home through foreclosure for financial reasons related to the pandemic. Renters, however, have a constant fear of eviction even during the best of times [27], making the shaky economic environment surrounding the pandemic perhaps exponentially more taxing on their mental health. Likewise, with one additional person added to the household during pandemics, the odds of having mental health problems declined. Contrary to the belief that additional people within the household environment caused stress during the pandemic era, the results suggested that increased household size helped improve social interaction and companionship, which ultimately had beneficial effects on householders’ mental health and may have ultimately served as a bonding force for families during an uncertain time.

For individuals with annual household incomes between $25,000 and $49,999, the odds of having mental health problems were 1.019 times those with incomes less than $25,000. The odds diminish as the income category gets higher. Hence, improving residents’ income may help to mitigate mental health problems in general and during the pandemic in particular. The odds of having mental health problems were different between non-Whites and Whites. For instance, the odds of experiencing mental health problems for Blacks were 0.283 times and Asians were 0.121 times that of Whites. Level of education was positively and significantly associated with mental health problems, with those with some college or an associate’s degree were 1.188 times more than those with less than high school level education. Meanwhile, those with a graduate degree were 1.503 times more compared to those with less than a high school degree. The odds of having mental health problems for those who were never married and those who were widowed, divorced, or separated were 1.221 times and 1.197 times than those currently married, respectively. The odds of having mental health problems for females were 1.740 times that of males, suggesting females are more vulnerable during a pandemic. The odds of having mental health problems increases by 1.028 units if the individual birth year increases by one year, suggesting that younger people are susceptible to mental health issues due to pandemics.

The status of one’s job was also a flash point for many within the survey. For those who did not lose their employment, the odds of having mental health problems were 0.699 times that of those who lost their work during this time. Expectations also played a role; those who did not expect to lose their job had the odds of having mental health problems 0.561 times those who expected job loss due to pandemics. This finding was not unexpected, given that the anxiety involved with a tenuous work situation could possibly be more acute than a situation in which the job loss has already occurred. Furthermore, the odds of having mental health problems for individuals not engaged in any work, were approximately 1.114 times than those engaged in such activities.

Food supply also proved to be a reliable indicator of one’s overall anxiety levels. The findings suggested that household’s food sufficiency was significantly associated with the respondent’s mental health status. For example, the odds of having mental health problems for individuals not having sufficient food availability during the pandemic were 2.904 times than those who had sufficient food at their disposal. Similarly, the odds of having mental health problems for individuals with good health status were 2.142 and individuals with fair or poor health statuses were 3.907 times those with excellent or very good health statuses. Further, the findings suggested that a decrease in relative food sufficiency (a derived variable) from pre-pandemic levels to levels during the pandemic leads to increased odds of having mental health problems. This finding was understandable given not only the lower amount of food that exists in the home currently relative to what supply normally is, but also the added anxiety involved with venturing into a public space such as a grocery store during the risky times. Combined with the food and supply shortages that have occurred at various times in stores since March 2020, it would be hard not to imagine these series of events negatively affecting on one’s mental health.

Overall health care has a logical connection with one’s mental health condition, and the results confirm this connection. The odds of having mental health problems for an individual without delayed medical care were 0.475 times those who could not access such medical care, suggesting improving access to health care improves people’s mental health condition during pandemics. Even medical care that is not related to pandemic-caused mental anguish, with those experiencing delays in receiving such care being 0.711 times more likely than those who did not have access to healthcare without such delays. There were slightly lower (yet significant) odds of experiencing mental health problems for those not having health insurance coverage compared to those having health insurance coverage.

### 3.2. Mental Health and Residential Environment: Building Type and Number of Household Member

For building type, a new model was created that incorporated only Weeks 13–19 data, since the previous twelve weeks did not initially capture this data point. The model has the same set of controls (sociodemographic variables and general health-related questions) and week-fixed effects as in Table 5. The new model (shown in Table 6) substitutes the tenure variable (whether the person is a renter or homeowner) with the building type where they reside. The findings suggested that the higher the density of the building, the greater were the chances of experiencing mental health problems. Other covariates were significant, as in Table 5.

### 3.3. Interaction Effects: Number of Individuals within Households and Housing Tenure and Income on Mental Health

We explored the interaction effects between the number of individuals within households and the type of building, and number of people within household and income, on mental health status (Table 7). The interaction model includes the same set of explanatory or control variables and week-fixed effects as they are in the baseline model (Table 5). The interaction between tenure (renters) and the number of individuals within the household was negative and significant, indicating that increasing the number of individuals within renters’ households was associated with lower mental health concerns during the pandemic. However, the interaction effects between the number of individuals within household and all categories of incomes (Income: $25,000 to Income: $150,000 and above) were all positive and significant, when compared against the lowest income category (Income: less than $25,000). Hence, increasing the number of individuals within the household regardless of household income was associated with greater mental health concerns.

## 4. Conclusions

As can be expected of a longitudinal survey of this size, several data points and results were of interest. First, the size and scope of the mental health problem cannot be understated. Whether the respondent felt down, worried, anxious, or uninterested, knowing that 60–67 percent of those surveyed expressed such mental health concerns amplifies the hidden public health concern that many people feared likely to occur. Renters fared worse than homeowners from a mental health perspective may have been largely due to a lack of control within their housing situations. This could be from an inability to adhere to social distancing guidelines within a dense environment to having others within the property management hierarchy setting the rules for its occupants. Therefore, a case could be made that housing satisfaction could suffer as a result of being relatively deprived of previously enjoyed amenities. For those renters, the size of the building also seemed to have a detrimental effect on their mental health. Specifically, the more densely populated the building, the more likely that mental health issues would occur. This could be largely the result of an inability to socially distance from other residents in higher-density environments. It is also possible that this result is an offshoot of well-established research showing that neighboring trends are declining in the U.S. [28], and that dense environments force people to interact with neighbors more often, potentially causing stress in the process. Finally, the addition of family members or extra household members served as a positive influence on one’s mental health. Contrary to the fears about family members constantly disturbing others once lockdowns were announced and people were forced to work from home while schools were also closed, the presence of others seemed to have a salient effect on householders’ mental health. This reinforces the notion that crises can be a rallying point for families, particularly larger ones. However, the single-person households are likely to suffer the most, which reinforces existing literature on the connection of loneliness and mental health problems among all age groups [29,30].

One of the limitations of this study was the finite period in which the data were analyzed. Whereas the pandemic is still ongoing and has been part of lives for over one year, this particular data set is only a short snapshot of time during the pandemic period. It can be argued that continued lockdowns and economic anxiety due to the COVID-19 outbreak has ebbed and flowed throughout the year that the country has been in some form of lockdown. It is also fair to point out that property managers have also had their own mental health concerns. The quality of customer care provided by this important part of the housing services community cannot be understated, and those who do not provide the proper amount of empathy to their apartment residents can help exacerbate an already growing problem.

The time covered during the Household Pulse Survey also serves as somewhat of a limitation. While the total number of periods is stated as 19 weeks, the actual timing of the survey needs more explanation. First, the survey began in late April, which was several weeks after the COVID-19 pandemic began and at least five weeks before nationwide lockdowns began. Consequently, the research community lost the opportunity to see how mental health problems compared to those that occurred during the early days of the pandemic. It is also noteworthy that the word “week” is somewhat of a misnomer. During the first phase of the Household Pulse Survey (weeks 1–12), the survey followed a weekly pattern, but subsequent Phases 2 and 3 extended the time frame to a two-week survey period. Finally, there was nearly a month-long pause between Phases 1 and 2, which prevented a truly longitudinal path of response patterns to be established. The delayed start-up of Week 13 created a lag that outside forces may have contributed to some of the mental health spikes shown during that week. This interregnum period between 21 July and 19 August included several events that caused much tumult and uncertainty within the country, including a bitterly divided Presidential campaign and the protests that occurred as fallout from the George Floyd killing by a police officer in Minneapolis. An unexpected surge of positive virus cases during the summer months only heightened the national anxiety.

The time period representing this study is noteworthy in that it possibly only represents one stage of a multi-layered complexity of mental health concerns brought on by the pandemic. The 19-week period covered in this research preceded the previously mentioned moratorium on evictions, which would be considered a period of heightened anxiety of whether the renter household in question would be able to meet its payment obligations going forward. The eviction moratorium would have helped to alleviate some of those concerns, but the threat of lifting such bans can bring on additional stress. At the time of this writing, it seemed clear that the ban on eviction would soon be coming to an end [31]. As a result, continuing this analysis a year into the COVID-19 era may have some merit due to the economic anxieties that persist. Coupled with a drop in rental availability in some areas [32], this creates even more anxiety among households looking to make a move in the post-pandemic era.

Property management personnel have also learned a few things along the way related to the pandemic and its effects on their residents. Owners and operators of rental properties have seen the need to incorporate addressing mental health as part of the industry’s overall push toward sustainability. Therefore, a certification program toward that end is now well underway within the industry, which serves as a seal of approval for operators of healthy buildings, providing third-party verification that extensive protocols and processes are in place [33]. Despite having been put in place only three years ago, growth in applications for this certification has exceeded 120 per cent [34]. This type of push for a comprehensive wellness program for both multifamily buildings and their residents may help to allay mental health concerns in the future, whether during a pandemic or during normal times. For this reason, apartment dwellers may have more access to resources than their single-family counterparts.

Ultimately, it is unclear what the COVID-19 crisis will mean for the future of the rental real estate industry from the demand side. Some futurists believe that the long-term impacts on the real estate industry may be harsh, with people eschewing the urban life for the perceived safety of rural locales [35]. If this trend were to take shape, it would reinforce an established societal housing norm of homeownership over rental housing [36]. Such a projection, however, would cut against the powerful forces that demographers point to in the constant push for urbanization as part of the larger demographic transition over time [37]. Nonetheless, the COVID-19 episode will likely have lasting mental health effects for Americans and the world going forward, no matter where they live.

## Figures and Tables

**Table 1:** Description of Household Pulse Survey variables.

Variable	Description	Question Wording	Range
TBIRTH_YEAR	Year of birth	What year were you born? Please enter a number.	1932:2002
EGENDER	Gender (1) Male (2) Female	Are you … Select only one answer.	1:2
RRACE	Race (1) White, alone (2) Black, alone (3) Asian, alone (4) Any other race alone, or race in combination	What is your race? Please select all that apply.-Selected Choice-White	1:4
EEDUC	Educational attainment (1) Less than high school (2) Some high school (3) High school graduate or equivalent (for example GED) (4) Some college, but degree not received or is in progress (5) Associate’s degree (for example AA, AS) (6) Bachelor’s degree (for example BA, BS, AB) (7) Graduate degree (for example master’s, professional, doctorate)	What is the highest degree or level of school you have completed? Select only one answer.	1:7
MS	Marital status (1) Now married (2) Widowed (3) Divorced (4) Separated (5) Never married	What is your marital status? Select only one answer.	1:5
THHLD_NUMPER	Total number of people in household	How many total people—adults and children–currently live in your household, including yourself? Please enter a number.	1:10
WRKLOSS	Recent household job loss (1) Yes (2) No	Have you, or has anyone in your household experienced a loss of employment income since 13 March 2020? Select only one answer	1:2
EXPCTLOSS	Expected household job loss (1) Yes (2) No	Do you expect that you or anyone in your household will experience a loss of employment income in the next 4 weeks because of the coronavirus pandemic? Select only one answer	1:2
ANYWORK	Employment status for last 7 days (1) Yes (2) No	Now we are going to ask about your employment. In the last 7 days, did you do ANY work for either pay or profit? Select only one answer.	1:2
PRIFOODSUF	Food sufficiency prior to 13 March 2020 (1) Enough of the kinds of food (I/we) wanted to eat (2) Enough, but not always the kinds of food (I/we) wanted to eat (3) Sometimes not enough to eat (4) Often not enough to eat	Getting enough food can also be a problem for some people. Which of these statements best describes the food eaten in your household before 13 March 2020? Select only one answer.	1:4
HLTHSTATUS	General health status (1) Excellent (2) Very good (3) Good (4) Fair (5) Poor	Would you say your health in general is excellent, very good, good, fair, or poor? Select only one answer.	1:5
DOWN	Frequency of feeling depressed over previous 7 days (1) Not at all (2) Several days (3) More than half the days (4) Nearly every day -99) Question seen but category not selected -88) Missing/Did not report	Over the last 7 days, how often have you been bothered by ... feeling down, depressed, or hopeless? Would you say not at all, several days, more than half the days, or nearly every day? Select only one answer.	1:4
ANXIOUS	Frequency of anxiety over previous 7 days (1) Not at all (2) Several days (3) More than half the days (4) Nearly every day -99) Question seen but category not selected -88) Missing/Did not report	Over the last 7 days, how often have you been bothered by the following problems ... Feeling nervous, anxious, or on edge? Would you say not at all, several days, more than half the days, or nearly every day? Select only one answer.	1:4
WORRY	Frequency of worry over previous 7 days (1) Not at all (2) Several days (3) More than half the days (4) Nearly every day -99) Question seen but category not selected -88) Missing/Did not report	Over the last 7 days, how often have you been bothered by the following problems ... Not being able to stop or control worrying? Would you say not at all, several days, more than half the days, or nearly every day? Select only one answer.	1:4
INTEREST	Frequency of feeling depressed over previous 7 days (1) Not at all (2) Several days (3) More than half the days (4) Nearly every day -99) Question seen but category not selected -88) Missing/Did not report	Over the last 7 days, how often have you been bothered by ... having little interest or pleasure in doing things? Would you say not at all, several days, more than half the days, or nearly every day? Select only one answer.	1:4
HLTHINS1	Health Insurance Coverage- Insurance through a current or former employer or union (through yourself or another family member) (1) Category marked- Insurance through a current or former employer or union (through yourself or another family member) (2) Category marked “No” -99) Question seen but category not selected -88) Missing/Did not report	Are you currently covered by any of the following types of health insurance or health coverage plans? Mark Yes or No for each.-Insurance through a current or former employer or union (through yourself or another family member)	1:2
DELAY	Delayed medical care in last 4 weeks due to pandemic (1) Yes (2) No -99) Question seen but category not selected -88) Missing/Did not report	At any time in the last 4 weeks, did you DELAY getting medical care because of the coronavirus pandemic? Select only one answer.	1:2
NOTGET	Delayed medical care for something not related to pandemic (1) Yes (2) No -99) Question seen but category not selected -88) Missing/Did not report	At any time in the last 4 weeks, did you need medical care for something other than coronavirus, but DID NOT GET IT because of the coronavirus pandemic? Select only one answer.	1:2
TENURE	Housing owned or rented (1) Owned free and clear? (2) Owned with a mortgage or loan (including home equity loans)? (3) Rented? (4) Occupied without payment of rent? -99) Question seen but category not selected -88) Missing/Did not report	Is your house or apartment? Select only one answer.	1:4
INCOME	Total household income (before taxes) (1) Less than $25,000 (2) $25,000–$34,999 (3) $35,000–$49,999 (4) $50,000–$74,999 (5) $75,000–$99,999 (6) $100,000–$149,999 (7) $150,000–$199,999 (8) $200,000 and above -99) Question seen but category not selected -88) Missing/Did not report	In 2019 what was your total household income before taxes? Select only one answer.	1:8
WEEK	Week of interview	-	1:19
	1: 19		
LIVQTR	Building Type (1) A mobile home (2) A one-family house detached from any other house (3) A one-family house attached to one or more houses (4) A building with 2 apartments (5) A building with 3 or 4 apartments (6) A building with 5 to 9 apartments (7) A building with 10 to 19 apartments (8) A building with 20 to 49 apartments (9) A building with 50 or more apartments (10) Boat, RV, van, etc. -99) Question seen but category not selected -88) Missing/Did not report	Which best describes this building? Include all apartments, flats, etc., even if vacant. Select only one answer.	1:10

**Table 2:** Household Pulse Survey responses per week.

Week	Total
1	68,892
2	39,264
3	119,014
4	90,237
5	93,674
6	74,835
7	67,901
8	99,339
9	90,708
10	83,457
11	83,950
12	79,253
13	92,344
14	93,945
15	84,879
16	81,571
17	75,466
18	49,597
19	60,815

**Table 3:** Descriptive statistics of Household Pulse Survey respondents.

Variables	%
Age of Householders	
≤20	0.7
21–30	8.0
31–40	18.6
41–50	19.2
51–60	19.1
61–70	20.0
71–80	11.9
>80	2.5
Gender	
Male	40.6
Female	59.4
Race	
White alone	82.4
Black alone	8.0
Asian alone	4.8
Any other race	4.9
Education level	
Less than high school	0.6
Some high school	1.5
High school Graduate/GED	11.8
Some college	21.6
Associate’s degree	10.5
Bachelor’s degree	29.0
Graduate degree	25.1
Marital status	
Now married	57.5
Widowed	4.9
Divorced	15.2
Separated	1.9
Never married	19.8
Total household number	
1	16.8
2	37.0
3	17.3
4	15.8
5	7.4
6	3.1
7 or more	2.6

**Table 4:** (**A**) Weekly percent distribution of mental health concerns during pandemic, overall sample and by tenure status. (**B**) Mental health concerns during pandemic, by living arrangements.

**Week**	**Mental Health Status (*N* = 1,530,047)**		**Mental Health Status by TENURE: Homeowner (*N* = 1,107,306)**		**Mental Health Status by TENURE: Renter (*N* = 369,170)**	
	**No (*N* = 426,577)**	**Yes (*N* = 1,103,470)**	**No (*N* = 344,632)**	**Yes (*N* = 762,674)**	**No (*N* = 67,588)**	**Yes (*N* = 301,582)**
**A**						
1	33.27%	66.73%	30.62%	69.38%	19.49%	80.51%
2	33.82%	66.18%	32.34%	67.66%	19.68%	80.32%
3	33.91%	66.09%	31.45%	68.55%	18.73%	81.27%
4	33.13%	66.87%	33.99%	66.01%	20.21%	79.79%
5	34.08%	65.92%	33.40%	66.60%	19.50%	80.50%
6	34.17%	65.83%	33.19%	66.81%	19.12%	80.88%
7	35.69%	64.31%	31.18%	68.82%	18.45%	81.55%
8	36.24%	63.76%	31.14%	68.86%	17.60%	82.40%
9	37.48%	62.52%	29.78%	70.22%	16.97%	83.03%
10	37.87%	62.13%	29.33%	70.67%	16.69%	83.31%
11	38.79%	61.21%	28.31%	71.69%	15.58%	84.42%
12	39.52%	60.48%	27.65%	72.35%	16.17%	83.83%
13	35.41%	64.59%	32.36%	67.64%	19.36%	80.64%
14	35.00%	65.00%	32.60%	67.40%	19.92%	80.08%
15	35.63%	64.37%	32.29%	67.71%	19.37%	80.63%
16	35.30%	64.70%	32.48%	67.52%	19.72%	80.28%
17	36.21%	63.79%	31.55%	68.45%	18.48%	81.52%
18	38.00%	62.00%	29.14%	70.86%	16.35%	83.65%
19	39.49%	60.51%	26.22%	73.78%	15.34%	84.66%
**Week**	**Mental Health Status by** **LIVQTR = Low Density** **(*N* = 407,352)**		**Mental Health Status by** **LIVQTR = Medium Density (*N* = 45,258)**		**Mental Health Status by** **LIVQTR = High Density** **(*N* = 50,834)**	
	**No** **(*N* = 122,792)**	**Yes** **(*N* = 284,560)**	**No (*N* = 8832)**	**Yes (*N* = 36,426)**	**No (*N* = 8980)**	**Yes (*N* = 41,854)**
**B**						
13	31.19%	68.81%	19.89%	80.11%	19.51%	78.05%
14	31.48%	68.52%	20.42%	79.58%	21.24%	78.76%
15	31.18%	68.82%	19.87%	80.13%	21.63%	78.37%
16	31.33%	68.67%	20.99%	79.01%	21.04%	78.96%
17	30.36%	69.64%	19.04%	80.96%	21.41%	78.59%
18	28.00%	72.00%	18.00%	82.00%	17.94%	82.06%
19	25.01%	74.99%	16.84%	83.16%	17.76%	82.24%

**Table 5:** Logistic regression—mental health with housing tenure and number of people in the household as predictor variable.

Variables	Coefficient Estimate	Odds Ratio	Std. Error	z Value	Pr (>|z|)
Tenure: Owned with a mortgage	0.19	1.209	0.01	36.140	0.00
Tenure: Occupied without payment of rent	0.29	1.336	0.01	42.010	0.00
Number of people in household	−0.03	0.970	0.00	−17.080	0.00
Income: $25,000–$49,999	0.01	1.010	0.01	1.3300	0.18
Income: $50,000–$99,999	−0.06	0.942	0.01	−6.1000	0.00
Income: $100,000–$150,000	−0.09	0.914	0.01	−8.9300	0.00
Income: $150,000 and above	−0.11	0.896	0.01	−10.080	0.00
Race: Black alone	−0.34	0.712	0.01	−39.550	0.00
Race: Asian alone	−0.12	0.887	0.01	−12.240	0.00
Race: Any other race	−0.08	0.923	0.01	−6.9200	0.00
Education: High school graduate	−0.01	0.990	0.02	−0.6300	0.53
Education: Some college	0.17	1.185	0.02	8.9800	0.00
Education: Graduate degree	0.41	1.507	0.02	21.840	0.00
Marital Status: Never married	0.21	1.234	0.01	30.650	0.00
Marital Status: Widowed, divorced, separated	0.17	1.185	0.01	29.170	0.00
Gender: Female	0.55	1.733	0.00	130.03	0.00
Year of birth	0.03	1.030	0.00	144.06	0.00
Recent household job loss = No	−0.35	0.705	0.01	−63.720	0.00
Expected household job loss = No	−0.57	0.566	0.01	−84.520	0.00
Employment status = No	0.12	1.127	0.01	24.530	0.00
Household food sufficiency = No	0.81	2.248	0.02	41.630	0.00
General health status = Good	0.76	2.138	0.01	146.09	0.00
General health status = Fair/Poor	1.35	3.857	0.01	161.39	0.00
Delayed medical care due to pandemic = No	−0.75	0.472	0.01	−129.79	0.00
Delayed medical care something not related to pandemic = No	−0.34	0.967	0.01	−52.320	0.00
Health insurance = No	−0.03	0.970	0.01	−5.5100	0.00
Relative Food Sufficiency	0.25	1.284	0.03	7.8700	0.00
Intercept	−50.86	0.000	0.36	−140.52	0.00
Week-specific unobserved heterogeneities	Included				
Number of observations	1,476,476				

**Table 6:** Logistic regression—mental health with building type and number of people in the household as predictor variable.

Variables	Coefficient Estimate	Odds Ratio	Std. Error	z Value	Pr(z)
Building with 2 apartments	0.054	1.056	0.017	3.120	0.002
Building with 3 or 4 apartments	0.167	1.182	0.023	7.421	0.000
Building with 5 to 9 apartments	0.171	1.187	0.023	7.397	0.000
Building with 10 to 19 apartments	0.164	1.178	0.024	6.684	0.000
Building with 20 to 49 apartments	0.251	1.285	0.026	9.800	0.000
Building with 50 or more apartments	0.187	1.206	0.019	10.060	0.000
Number of people in household	−0.034	0.966	0.003	−11.677	0.000
Income: $25,000–$49,999	0.008	1.008	0.016	0.500	0.617
Income: $50,000–$99,999	−0.037	0.964	0.016	−2.263	0.024
Income: $100,000–$150,000	−0.062	0.940	0.018	−3.462	0.001
Income: $150,000 and above	−0.057	0.945	0.018	−3.118	0.002
Race: Black, alone	−0.355	0.701	0.015	−24.037	0.000
Race: Asian, alone	−0.284	0.753	0.016	−17.330	0.000
Race: Any other race)	−0.109	0.897	0.018	−5.979	0.000
Education: High school graduate	0.057	1.058	0.033	1.721	0.085
Education: Some college	0.264	1.303	0.032	8.300	0.000
Education: Graduate degree	0.568	1.765	0.032	17.792	0.000
Marital status: Never married	0.191	1.211	0.012	16.193	0.000
Marital status: Widowed, divorced, separated	0.172	1.188	0.010	17.238	0.000
Gender: Female	0.584	1.792	0.007	80.635	0.000
Year of birth	0.027	1.027	0.000	87.273	0.000
Recent household job loss = No	−0.403	0.669	0.009	−44.294	0.000
Expected household job loss = No	−0.567	0.567	0.013	−44.856	0.000
Employment status = No	0.145	1.155	0.009	16.475	0.000
Household food sufficiency = No	1.111	3.036	0.042	26.225	0.000
General health status = Good	0.768	2.155	0.009	88.047	0.000
General health status = Fair/Poor	1.385	3.994	0.014	98.603	0.000
Delayed medical care due to pandemic = No	−0.912	0.402	0.011	−86.484	0.000
Delayed medical care something not related to pandemic = No	−0.395	0.674	0.013	−31.322	0.000
Health insurance = No	−0.018	0.982	0.009	−2.068	0.039
Decreased Food Sufficiency	0.284	1.328	0.052	5.414	0.000
Intercept	−51.150	0.000	0.604	−84.692	0.000
Week-specific unobserved heterogeneities	Included				
Number of observations	468,459				

**Table 7:** Exploring interaction effects between number of individuals within households and housing tenure and income.

Variables	Coefficient Estimate	Odds Ratio	Std. Error	z Value	Pr (>|z|)
Tenure: Renter & Number of people in household	−0.037	0.96	0.004	−10.472	0.000
Income: $25,000–$49,999 & Number of people in household	0.023	1.02	0.023	4.330	0.000
Income: $50,000–$99,999 & Number of people in household	0.019	1.02	0.005	3.687	0.000
Income: $100,000–$150,000 & Number of people in household	0.020	1.02	0.006	3.526	0.000
Income: $150,000 and above & Number of people in household	0.014	1.01	0.006	2.419	0.016
All other covariates	Included				
Week-specific unobserved heterogeneities	Included				
Number of observations	1,476,476				

## Data Availability

Publicly available datasets were analyzed in this study. The data can be found here: https://www.census.gov/programs-surveys/household-pulse-survey/datasets.html (accessed on 20 January 2021).
